# Factors associated with the health and reproductive autonomy of Quilombola women in Brazil

**DOI:** 10.17533/udea.iee.v42n1e10

**Published:** 2024-04-29

**Authors:** Gabriela Cardoso Moreira Marques, Silvia Lucia Ferreira, Eliana do Sacramento de Almeida, Paloma Leite Diniz Farias, Sânzia Bezerra Ribeiro, Edméia de Almeida Cardoso Coelho

**Affiliations:** 1 Registered Nurse. PhD. Assistant Professor, Nursing Course at the Universidade do Estado da Bahia, Guanambi, Bahia, Brasil. Email: gmarques.vc@gmail.com Universidade do Estado da Bahia Universidade do Estado da Bahia Guanambi Bahia Brazil gmarques.vc@gmail.com; 2 Registered Nurse. PhD. Full Professor, Escola de Enfermagem da Universidade Federal da Bahia, Salvador, Bahia, Brazil. Email: silvialf100@gmail.com Universidade Federal da Bahia Escola de Enfermagem Universidade Federal da Bahia Salvador Bahia Brazil silvialf100@gmail.com; 3 Registered Nurse. PhD candidate. Assistant Professor, Nursing Course at the Universidade do Estado da Bahia, Senhor do Bonfim, Bahia, Brazil. Email: elianadosacramento@hotmail.com Universidade do Estado da Bahia Universidade do Estado da Bahia Senhor do Bonfim Bahia Brazil elianadosacramento@hotmail.com; 4 Bachelor of Laws. PhD candidate. Judicial Analyst at the Tribunal de Justiça do Estado da Paraíba, Brazil. Email: palomaldf@gmail.com Tribunal de Justiça do Estado da Paraíba Tribunal de Justiça do Estado da Paraíba Brazil palomaldf@gmail.com; 5 Physiotherapist. PhD candidate. Professor, Physiotherapy Course at UNIAENE-FADBA - Adventist University Center of Northeast, Cachoeira, Bahia, Brazil. Email: sanziar@gmail.com Adventist University Center of Northeast Physiotherapy Course at UNIAENE FADBA Adventist University Center of Northeast Cachoeira Bahia Brazil sanziar@gmail.com; 6 Registered Nurse. PhD. Full Professor, Escola de Enfermagem da Universidade Federal da Bahia, Salvador, Bahia, Brazil. Email: edmeiaacardoso3@gmail.com Universidade Federal da Bahia Escola de Enfermagem Universidade Federal da Bahia Salvador Bahia Brazil edmeiaacardoso3@gmail.com

**Keywords:** women, Quilombola communities, reproductive health, socioeconomic survey, personal autonomy, nursing., mujeres, quilombola, salud reproductiva, encuesta socioeconómica, autonomía personal, enfermería., mulheres, quilombola, saúde reprodutiva, enquete socioeconômica, autonomia pessoal, enfermagem.

## Abstract

**Objective.:**

To verify the association between reproductive autonomy and sociodemographic, sexual, and reproductive characteristics in Quilombola women (a term indicating the origin of politically organized concentrations of Afro-descendants who emancipated themselves from slavery).

**Methods.:**

Cross-sectional and analytical study with 160 women from Quilombola communities in the southwest of Bahia, Brazil. Data were collected using the Reproductive Autonomy Scale and the questionnaire from the National Health Survey (adapted).

**Results.:**

Out of the 160 participating women, 91.9% declared themselves as black, one out of every three were aged ≤ 23 years, 53.8% were married or had a partner, 38.8% had studied for ≤ 4 years, over half (58.1%) were unemployed, only 32.4% had a monthly income > R$ 430 (80 US dollars), 52.5% had their first menstruation at the age of 12, 70.7% had not accessed family planning services in the last 12 months, and over half used some method to avoid pregnancy (59.0%). The women had a high level of reproductive autonomy, especially in the "Decision-making" and "Freedom from coercion" subscales with a score of 2.53 and 3.40, respectively. A significant association (*p*<0.05) was found between the "Total reproductive autonomy" score and marital status, indicating that single or unpartnered women had higher autonomy compared to married or partnered women.

**Conclusion.:**

The association of social determinants of health such as marital status, education, and age impacts women's reproductive choices, implying risks for sexual and reproductive health. The intergenerational reproductive autonomy of Quilombola women is associated with sociodemographic and reproductive factors.

## Resumen

**Objetivo.:**

Verificar la asociación entre autonomía reproductiva y características sociodemográficas, sexuales y reproductivas en mujeres *quilombolas* (término que indica procedencia de concentraciones de afrodescendientes políticamente organizadas que se emanciparon de la esclavitud).

**Métodos.:**

Estudio transversal y analítico con 160 mujeres de comunidades quilombolas del sudoeste de Bahía, Brasil. Los datos fueron recolectados utilizando la Escala de Autonomía Reproductiva y el cuestionario de la Encuesta Nacional de Salud (adaptado).

**Resultados.:**

De las 160 mujeres participantes 91.9% se declararon negras, una de cada tres tenía edad ≤ 23 años, 53.8% estaban casada o tenían pareja, 38.8% había estudiado por ≤ 4 años, más de la mitad (58.1%) no trabajaba, solo 32.4% tenía renta > R$ 430 mensual (87 $US dólares), el 52.5% tuvo la primera menstruación a los 12 años, 70.7% no había acudido a servicios de planificación familiar en los últimos 12 meses y más de la mitad usaba algún método para evitar embarazo (59%). Las mujeres tuvieron un alto nivel de autonomía reproductiva, especialmente en las subescalas "Toma de decisiones" y "Ausencia de coerción" con una puntuación de 2.53 y 3.40, respectivamente. Se encontró asociación significativa (*p*<0.05) entre la puntuación de "Autonomía reproductiva total" con el estado civil, indicando el análisis que las mujeres solteras o sin pareja tenían mayor autonomía en comparación con las casadas o con pareja.

**Conclusión.:**

La asociación de determinantes sociales de la salud como el estado civil, la escolaridad y la edad interfieren en las opciones reproductivas de las mujeres, implicando riesgos para la salud sexual y reproductiva. La autonomía reproductiva intergeneracional de las mujeres quilombolas está asociada a factores sociodemográficos y reproductivos.

## Resumo

**Objetivo.:**

Verificar a associação entre a autonomia reprodutiva e características sociodemográficas, sexuais e reprodutivas em mulheres quilombolas (termo que indica a origem de concentrações politicamente organizadas de pessoas de ascendência africana que se emanciparam da escravatura).

**Métodos.:**

Estudo transversal e analítico com 160 mulheres (80 mães e 80 filhas) de comunidades quilombolas no sudoeste baiano, no Brasil. Os dados foram construídos através da aplicação da Escala de Autonomia Reprodutiva e do questionário da Pesquisa Nacional de Saúde (adaptado).

**Resultados.:**

das 160 mulheres participantes 91.9% se autodeclararam negra, a maioria com idade ≤ 23 anos (35.6%), 53.8% são casadas ou com companheiro, 38.8% com estudos ≤ 4 anos, mais da metade (58.1%) não trabalham, apenas 32.4% têm renda > R$ 430, a maioria teve a primeira menstruação até os 12 anos de idade (52.5%), não participou de grupo de planejamento familiar nos últimos 12 meses (70.7%), mais da metade utilizava método para evitar a gravidez (59%). Apresentaram elevada autonomia reprodutiva, com destaque para as subescalas “Tomada de decisão” e “Ausência de coerção” medindo 2.53 e 3.40, respectivamente. Encontrou-se associação significativa (*p<*0.05) entre o escore de “Autonomia reprodutiva total” e estado conjugal, com a análise indicando que mulheres solteiras ou sem companheiro apresentaram maior autonomia, comparadas às mulheres casadas ou com companheiro.

**Conclusão.:**

A associação dos determinantes sociais de saúde como estado civil, menarca, escolaridade e idade interferem nas escolhas reprodutivas das mulheres, implicando em riscos à saúde sexual e reprodutiva. A autonomia reprodutiva intergeracional das mulheres quilombolas está associada a fatores sociodemográficos e reprodutivos.

## Introduction

The relationship a woman has with her sexual partner, the culture, and the context in which she lives influence her ability to achieve her reproductive intentions. Accordingly, the level of reproductive autonomy is shaped as these influences change, which can differ among women with multiple partners, women without partners, women living in a community with strong reproductive rights, and women living in a community where reproductive rights are not supported.^1^ Autonomy is considered essential for decision-making in various healthcare situations, from seeking and using care to choosing treatment options. Women's ability to take care of their health and utilize healthcare services appropriately may partly depend on their autonomy to make decisions.^2^

Women's reproductive decisions may be impacted by factors such as marital status, age, sociodemographic conditions, color and race, religion, occupation, geographic region, and education.^3^ Due to geographic distances, difficulty of access, lack of infrastructure in services, and scarcity of public policies, rural populations tend to be disadvantaged in healthcare.^4^ Reproductive autonomy is essential for a woman as it facilitates her ability to choose childbirth, abortion, or contraception without undue influence from men, healthcare providers, the government, the international development community, or religious doctrine.^5^ This autonomy may fluctuate within different relationships and cultural contexts, depending on the degree to which the partner or the surrounding community supports reproductive rights.^6^ Sexual and reproductive health interfaces with various themes such as sexuality experience, human rights, cultural and religious aspects, gender relations, as well as access to healthcare services, which requires special attention from the healthcare sector, as they affect the health and well-being of individuals and communities.^7^ Accordingly, Black Quilombola women are often deprived of their sexual and reproductive rights, resulting from difficulties in accessing healthcare services, low educational attainment, and few opportunities determined by their living conditions, in addition to geographical difficulties.

Contextualizing, historically, quilombos were characterized as places isolated both temporally and geographically, serving as hiding spots for enslaved people who had escaped. Individuals and even entire families who were kidnapped from their homeland, the African continent, were forced into various types of work, exploitation, and violence. Currently, Quilombola communities are present in almost the entire Brazilian territory and remain vibrant and active, contributing to the country's development through agriculture, cuisine, handicrafts, and various cultural expressions. They are social groups whose ethnic identity still distinguishes them from the rest of society, due to their organizational form, their relationship with other groups, and their political action.

To promote reproductive health for women and prevent the risk of unwanted pregnancy, the use of contraceptive methods is of paramount importance. Broader access to family planning, especially to contraceptive methods, can substantially reduce unwanted pregnancies and unsafe abortions.^8^ A woman's ability to act according to her intention to use contraceptives may depend on the desires and actions of her partner or other members of her family or community.^6^ The role of women as still being submissive regarding sexual matters and women's accountability regarding reproductive issues hinder dialogue with their partners and increase their vulnerability.^9^ The cultural and religious backgrounds of a particular community have a powerful influence on health-seeking behavior.^8^ We can better identify and understand the influence of interpersonal power on reproductive behaviors, which can inform strategies to prevent unwanted pregnancy.^6^

When compared to white women, Black women present a higher risk of illness and death. It is emphasized that socioeconomic inequalities and institutional racism contribute to the high vulnerability of Black women due to double discrimination, as they live in unequal gender and ethnic/racial conditions.^9^ In this context, nursing is extremely relevant, considering that it plays an important role in Primary Health Care, through actions of disease prevention, health promotion, and health education. Therefore, this study allows the expansion of knowledge on the subject and provides support for nurses to rethink their professional practice, break with the fragmentation of care, and develop a qualified, respectful, and culturally oriented approach.^10^

Reproductive autonomy in Quilombola women is still a little-explored topic in the literature, justifying the present study, which aims to verify the association between reproductive autonomy and sociodemographic, sexual, and reproductive characteristics in Quilombola women.

## Methods

This was a cross-sectional and analytical study conducted with 160 Quilombola women (80 mothers and 80 daughters), of reproductive age from 18 to 49 years of age, residing in 2020 in Quilombola communities in the municipality of Vitória da Conquista in the state of Bahia, from July 2019 to March 2020. This municipality is located in the center of the Southwest Bahia Identity Territory and has 23 certified Quilombolo remnant communities (CRQs) updated until Ordinance No. 118/2020, published in the Official Gazette of the Union (DOU) on July 20, 2020. However, this research was conducted in 9 Quilombola communities belonging to the municipality of Vitória da Conquista: Ribeirão do Paneleiro, Barrocas, Boqueirão, Sinzoca, Lagoa dos Patos, Laranjeiras, São Joaquim do Sertão, Lagoa Maria Clemência, and Lagoa de Melquíades.

This manuscript is an excerpt from the thesis entitled "Intergenerational reproductive autonomy in Quilombola women" presented to the Graduate Program in Nursing and Health linked to the Nursing School of the Federal University of Bahia. Due to the COVID-19 pandemic and logistical difficulties, it was not possible to conduct a population census involving all communities in this municipality. The impossibility of accessing the record of the number of families per Quilombola community also made it impossible to use a probabilistic sample. Therefore, it was decided to conduct the research in only nine communities, through a non-probabilistic convenience sample that included women who met the following inclusion criteria: Quilombola women of reproductive age from 18 to 49 years of age; mothers and daughters from a Quilombola community certified by the Palmares Cultural Foundation in the municipality of Vitória da Conquista, who authorized the visit for data collection and signed the consent form. Women (mothers and daughters) who did not reside in the community at the time, who had cognitive or psychiatric disorders that could hinder the understanding of the data collection instrument, and those who, for any reason, did not complete the interview were excluded.

In order to provide greater territorial representativeness, the municipality was divided into Axes (Axis 1 - Central Quadrant; Axis 2 - North Quadrant; Axis 3 - Center-West Quadrant; and Axis 4 - South Quadrant). Then, the nine communities were randomly selected, obeying the proportionality by axis, resulting in the selection of the following communities: Ribeirão do Paneleiro, Barrocas, Boqueirão, Sinzoca, Lagoa dos Patos, Laranjeiras, São Joaquim do Sertão, Lagoa Maria Clemência, and Lagoa de Melquíades.

The data collection was carried out through the application of two instruments during visits to the women's (mothers and daughters) homes, accompanied by Quilombola leaders from their respective communities. The first instrument, the adapted National Health Survey (*Pesquisa Nacional de Saúde* - PNS) questionnaire, was used to cover, with independent variables, Module A (sociodemographic characteristics - age, marital status, level of education, self-declared color/race, religion, occupation); Module R (women's health - health, preventive exams, reproductive history, family planning, and contraception); and Module S (prenatal care and childbirth assistance).

The second instrument is the Reproductive Autonomy Scale, which aims to assess a woman's ability to achieve her reproductive intentions. The Brazilian version was translated from English to Portuguese and culturally adapted, proving suitable for evaluating the reproductive autonomy of Brazilian women and showing reliability in application among rural working women and rural Quilombola women, demonstrating acceptable internal consistency and reproducibility. The scale consists of 14 items in three subscales: "Decision-making," which evaluates who decides on using a method to avoid pregnancy, when to have a baby, and about an unplanned pregnancy; "Freedom from coercion," which addresses whether the partner prevented, hindered, or pressured the woman regarding using any contraceptive method to avoid pregnancy; and "Communication," related to the woman's comfort level in discussing her reproductive choices with her partner.

For data analysis, descriptive statistical procedures such as absolute and relative frequencies, means, medians, standard deviations (SD), interquartile ranges (IQR), and minimum and maximum values were used. Data normality was tested using the Shapiro-Wilk and Kolmogorov-Smirnov tests, while homoscedasticity was tested using Levene’s test. Comparisons between two groups were performed using the Mann-Whitney test or Student's *t*-test for independent samples, whereas comparisons between three groups were made through the Kruskal-Wallis test (pairwise comparisons using the Mann-Whitney test) or one-way analysis of variance (ANOVA) (multiple comparisons using Tukey's test). The significance level adopted in the study was 5% (α = 0.05), and all analyses were performed using the IBM SPSS version 21.0 software.

This research was guided by the ethical precepts governing Resolution No. 466/2012 of the National Health Council. Data collection began after approval by the Ethics and Research Committee of the Federal University of Bahia (UFBA), CAAE: 14087019.1.0000.5531, approval opinion No. 3.448.011, dated 07/10/2019.

The study adhered to all principles of bioethics such as non-maleficence, beneficence, autonomy, justice, and equity. Free choice to participate or not in the study was guaranteed, respecting the individuality and autonomy, the possibility of withdrawing or refusing to answer any questions, and providing the consent form for signature by all participants.

## Results

The age of the 160 study participants ranged from 18 to 49 years (mean = 32.8; SD = 11.4). The main sociodemographic characteristics of the participants are presented in Table 1. Similar distribution was observed among age groups, marital status, years of education, and income. The majority of the sample consisted of Black women (91.9%), who did not have paid employment and were Catholic (85.6%). Most were aged ≤ 23 years (35.6%), 53.8% were married or in a relationship, 38.8% had ≤ 4 years of education, and over half (58.1%) were not employed, with only 32.4% having income > R$ 430, equivalent to US$ 80.22 at the time of the study.

**Table 1:** Distribution of the 160 study participants according to sociodemographic characteristics

Variable	*n*	%
Age group		
≤ 23 years	57	35.6
24 to 42 years	54	33.8
> 42 years	49	30.6
Ethnicity		
White	13	8.1
Black	147	91.9
Marital status		
Single or without partner	74	46.3
Married or with partner	86	53.8
Years of education		
≤ 4 years	62	38.8
5 to 9 years	49	30.6
> 9 years	49	30.6
Currently employed		
Yes	67	41.9
No	93	58.1
Monthly income		
≤ R$ 130 (U$ 24.2)	56	37.8
R$ 131 to R$ 430 (U$ 24.3 to U$ 80.2)	44	29.7
> R$ 430 (>U$ 80.2)	48	32.4
Religion		
Non-Catholic	23	14.4
Catholic	137	85.6

In Table 2, the distribution of participants according to their sexual and reproductive characteristics is presented. The majority of women experienced menarche by the age of 12 (52.5%), underwent cervical cytology screening in the previous two years (56.9%), never had a mammogram (83.0%), had sexual intercourse in the previous 12 months (87.5%), did not participate in a family planning group in the previous 12 months (70.7%), their partner did not participate in a family planning group (98.0%), used a method to prevent pregnancy (59.0%), had been pregnant at least once (80.6%), and had at least one childbirth.

**Table 2:** Distribution of study participants according to sexual and reproductive characteristics

Variable	*n*	%
Age group (*n*=158)		
≤ 23 years	83	52.5
24 to 42 years	37	23.4
> 42 years	38	24.1
Underwent cervical cytology screening in the last 2 years (*n*=160)	91	56.9
Underwent mammography screening (*n*=159)	132	83.0
Had sexual intercourse in the last 12 months (*n*=160)	140	87.5
Participated in a family planning group in the last 12 months (*n*=157)	46	29.3
Partner's participation in a family planning group (*n*=151)	3	2.0
Use of method to prevent pregnancy (*n*=156)	92	59.0
Has been pregnant (*n*=160)	129	80.6
Number of childbirths (*n*=160)		
None	31	19.4
1 to 2	63	39.4
> 2	66	41.2

The means, standard deviations, and minimum and maximum values of the reproductive autonomy scores are presented in Table 3. Overall, it is considered that the participants exhibited high reproductive autonomy, with the subscales that stood out with the highest proportional scores being "Decision-making" with a mean score of 2.53 (84.3% of the maximum score) and "Freedom from coercion" with a mean score of 3.40 (85.0% of the maximum score).

**Table 3:** Descriptive analysis of the reproductive autonomy scores of the 160 study participants, according to each subscale of the Reproductive Autonomy Scale

Subscale	Mean	Standard deviation	Minimum - Maximum
Decision-making	2.53	0.36	1.50 - 3.00
Freedom from coercion	3.40	0.57	1.60 - 4.00
Communication	3.10	0.60	1.80 - 4.00
Total	3.05	0.34	2.07 - 3.71

The association between the reproductive autonomy scores (subscales and total) and the sociodemographic characteristics of the sample is presented in Table 4. An association was found between the "total reproductive autonomy" score and marital status, meaning that single women or those without a partner exhibited higher autonomy (3.07) compared to married women or those with a partner (2.93). The scores of the three subscales (Decision-making, Freedom from coercion, and Communication) did not show an association with the sociodemographic characteristics studied. 

**Table 4:** Association between the reproductive autonomy scores and the sociodemographic characteristics of the 160 study participants

Variable	Decision-making	Freedom from coercion	Communication	Total
Age group				
≤ 23 years	2.50 (IQR = 0.63)	3.40 (IQR = 1.00)	3.00 (IQR = 0.80)	3.07 (IQR = 0.43)
24 to 42 years	2.50 (IQR = 0.56)	3.60 (IQR = 1.00)	3.00 (IQR = 0.85)	3.07 (IQR = 0.50)
> 42 years	2.50 (IQR = 0.63)	3.20 (IQR = 0.80)	3.00 (IQR = 0.60)	2.93 (IQR = 0.43)
*p*-value	0.345	0.471	0.436	0.340
Ethnicity				
White	2.75 (IQR = 0.63)	3.00 (IQR = 1.10)	2.80 (IQR = 0.50)	2.93 (IQR = 0.54)
Black	2.50 (IQR = 0.50)	3.40 (IQR = 1.00)	3.00 (IQR = 0.86)	3.00 (IQR = 0.50)
*p*-value	0.284	0.310	0.174	0.330
Marital status				
Single or without partner	2.50 (IQR = 0.75)	3.60 (IQR = 1.00)	3.00 (IQR = 0.80)	3.07 (IQR = 0.36)
Married or with partner	2.50 (IQR = 0.50)	3.20 (IQR = 1.00)	3.00 (IQR = 0.85)	2.93 (IQR = 0.57)
*p*-value	0.174	0.250	0.417	0.040
Years of education				
≤ 4 years	2.50 (IQR = 0.56)	3.50 (IQR = 1.00)	3.00 (IQR = 1.00)	3.00 (IQR = 0.52)
5 to 9 years	2.50 (IQR = 0.50)	3.40 (IQR = 1.00)	3.00 (IQR = 0.90)	2.93 (IQR = 0.54)
> 9 years	2.50 (IQR = 0.75)	3.40 (IQR = 0.80)	3.20 (IQR = 0.70)	3.07 (IQR = 0.43)
*p*-value	0.846	0.578	0.085	0.217
Currently employed				
Yes	2.50 (IQR = 0.50)	3.60 (IQR = 1.00)	3.00 (IQR = 1.00)	3.07 (IQR = 0.50)
No	2.50 (IQR = 0.75)	3.40 (IQR = 1.00)	3.00 (IQR = 0.90)	3.00 (IQR = 0.50)
*p*-value	0.840	0,850	0.303	0.314
Monthly income				
≤ R$ 130 (U$ 24.2)	2.50 (IQR = 0.75)	3.40 (IQR = 1.00)	3.10 (IQR = 1.00)	3.09 (SD = 0.33)
R$ 131 to R$ 430 (U$ 24.4 to U$ 80.2)	2.50 (IQR = 0.75)	3.40 (IQR = 1.20)	3.00 (IQR = 0.95	2.95 (SD = 0.39)
> R$ 430 (U$ 80.2)	2.50 (IQR = 0.50)	3.30 (IQR = 0.75)	3.00 (IQR = 0.60)	3.04 (SD = 0.28)
*p*-value	0.705	0.319	0.317	0.134
Religion				
Non-Catholic	2.50 (IQR = 0.75)	3.40 (IQR = 1.00)	3.20 (IQR = 1.00)	3.00 (IQR = 0.50)
Catholic	2.50 (IQR = 0.50)	3.40 (IQR = 1.00)	3.00 (IQR = 0.80)	3.00 (IQR = 0.50)
*p*-value	0.454	0.915	0.720	0.936

Legend: IQR, interquartile range; SD, standard deviation. Values accompanied by IQR represent medians and were compared using the Kruskal-Wallis test (age group, years of education, and monthly income) or the Mann-Whitney test (ethnicity, marital status, currently employed, and religion); values accompanied by SD represent means and were compared using one-way ANOVA.

Associations between the reproductive autonomy scores and the sexual and reproductive characteristics of the sample were also investigated (Table 5). There was an association between the "Freedom from coercion" and "Total reproductive autonomy" scores with age at first menstruation and the performance of mammography screening. The analyses indicated that women who had late menarche (after 13 years) and who had undergone mammography screening demonstrated lower autonomy in the "Freedom from coercion" and "Total reproductive autonomy" subscales compared to their peers. The "Decision-making" and "Communication" scores were not associated with the sexual and reproductive characteristics evaluated.

**Table 5:** Association between the reproductive autonomy scores and the sexual and reproductive characteristics of the 160 study participants

Variable	Decision-making	Freedom from coercion	Communication	Total
Age at first menstruation	Age at first menstruation	Age at first menstruation	Age at first menstruation	Age at first menstruation
≤ 12 years	2.50 (IQR = 0.75)	3.60^a^ (IQR = 1.00)	3.00 (IQR = 1.00)	3.08^a^ (SD = 0.34)
13 years	2.50 (IQR = 0.75)	3.60^a^ (IQR = 1.00)	3.00 (IQR = 0.80)	3.08^a^ (SD = 0.32)
> 13 years	2.50 (IQR = 0.50)	3.00^b^ (IQR = 0.60)	3.00 (IQR = 0.65)	2.91^b^ (SD = 0.34)
*p*-value	0.456	0.020	0.162	0.029
Underwent cervical cytology screening in the last 2 years				
Yes	2.50 (IQR = 0.50)	3.60 (IQR = 1.00)	3.00 (IQR = 1.00)	3.07 (SD = 0.36)
No	2.50 (IQR = 0.75)	3.40 (IQR = 0.80)	3.00 (IQR = 0.60)	3.01 (SD = 0.32)
*p*-value	0.376	0.419	0.195	0.311
Underwent mammography screening				
No	2.50 (IQR = 0.69)	3.60 (IQR = 1.00)	3.00 (IQR = 0.80)	3.07 (IQR = 0.50)
Yes	2.50 (IQR = 0.50)	3.00 (IQR = 0.60)	3.00 (IQR = 1.20)	2.78 (IQR = 0.50)
*p*-value	0.351	0.009	0.110	0.002
Had sexual intercourse in the last 12 months				
No	2.50 (IQR = 0.69)	3.20 (IQR = 0.80)	3.00 (IQR = 0.40)	3.00 (IQR = 0.32)
Yes	2.50 (IQR = 0.69)	3.50 (IQR = 1.00)	3.00 (IQR = 0.95)	3.00 (IQR = 0.57)
*p*-value	0.645	0.522	0.416	0.696
Participated in a family planning group in the last 12 months				
No	2.50 (IQR = 0.50)	3.60 (IQR = 1.00)	3.00 (IQR = 1.00)	3.07 (SD = 0.34)
Yes	2.50 (IQR = 0.50)	3.40 (IQR = 1.00)	3.00 (IQR = 0.65)	2.99 (SD = 0.36)
*p*-value	0.097	0.189	0.123	0.222
Partner's participation in a family planning group				
No	2.50 (IQR = 0.69)	3.40 (IQR = 1.00)	3.00 (IQR = 0.80)	3.00 (IQR = 0.55)
Yes	2.50 (IQR = -)	3.00 (IQR = -)	3.00 (IQR = -)	2.93 (IQR = -)
*p*-value	0.598	0.743	0.946	0.769
Use of method to prevent pregnancy				
No	2.50 (IQR = 0.50)	3.60 (IQR = 1.00)	3.00 (IQR = 0.80)	3.04 (SD =0.38)
Yes	2.50 (IQR = 0.75)	3.40 (IQR = 1.00)	3.00 (IQR =0.75)	3.05 (SD = 0.32)
*p*-value	0.285	0.735	0.956	0.957
Has been pregnant				
No	2.50 (IQR = 0.75)	3.40 (IQR = 1.00)	3.20 (IQR = 0.80)	3.11 (SD =0.29)
Yes	2.50 (IQR = 0.63)	3.60 (IQR = 1.00)	3.00 (IQR =0.60)	3.03 (SD =0.35)
*p*-value	0.366	0.951	0.071	0.277
Number of childbirths				
None	2.50 (IQR = 0.75)	3.40 (IQR = 1.00)	3.20 (IQR = 0.80)	3.00 (IQR =0.43)
1 to 2	2.50 (IQR = 0.50)	3.40 (IQR = 1.00)	3.00 (IQR =0.60)	3.00 (IQR =0.50)
> 2	2.50 (IQR = 0.75)	3.60 (IQR = 1.00)	3.00 (IQR =0.90)	3.00 (IQR = 0.57)
*p*-value	0.472	0.995	0.197	0.608

Legend: IQR, interquartile range; SD, standard deviation; -, IQR could not be calculated due to the small group size (*n* < 4). Values accompanied by IQR represent medians and were compared using the Kruskal-Wallis test (age at first menstruation and number of childbirths) or the Mann-Whitney test (underwent cervical cytology screening in the previous 2 years, underwent mammography screening, had sexual intercourse in the previous 12 months, participated in a family planning group in the previous 12 months, partner's participation in a family planning group, use of method to prevent pregnancy, and has been pregnant); values accompanied by SD represent means and were compared using one-way ANOVA (age at first menstruation) or Student’s *t*-test for independent samples (underwent cervical cytology screening in the previous 2 years, participated in a family planning group in the previous 12 months, use of method to prevent pregnancy, and has been pregnant). ^(a.b)^ Different letters indicate significant differences between groups (Mann-Whitney test or Tukey's test).

## Discussion

The subscale score for "Decision making" varied from 1.00 to 3.00, while for the subscales "Freedom from coercion" and "Communication," the score ranged from 1.00 to 4.00. The subscales that stood out with the highest proportional scores were "Decision making" and "Freedom from coercion." The score of 2.53 (84.33% of the maximum score) for "Decision Making" is closer to 3.00, which is the maximum score, just as "Freedom from coercion" with a score of 3.40 (85% of the maximum score) is closer to 4.00. However, in the "Communication" subscale, the score was 3.10 (77.55% of the maximum score), which is further from the maximum score of 4.00. 

The results indicated that Quilombola women showed high reproductive autonomy in the "Decision Making" and "Freedom from coercion" subscales. However, when compared to the study conducted with American women, it was found that the subscales showing greater reproductive autonomy were "Freedom from coercion" and "Communication." These results demonstrate that sociodemographic characteristics and other factors are associated with different levels of reproductive autonomy for each subscale among women in both studies.^1^ These findings partially align with a study conducted with American women; the Quilombola women in the study represent 91.9% of Black ethnicity and showed high reproductive autonomy in the "Decision Making" and "Freedom from coercion" subscales, whereas Black women in the American study were associated with lower levels of reproductive autonomy in the "Freedom from coercion" and "Communication" subscales. However, the Black women showed higher levels of reproductive autonomy in the decision-making subscale. 

The marital status of Quilombola women in the study can be considered a determining sociodemographic factor in reproductive autonomy, as the women who were single or without a partner showed higher overall reproductive autonomy compared to those women who were married or with a partner. However, in the study with American women, being married was associated with higher levels of autonomy in the "Communication" subscale and lower levels of autonomy in the "Decision making" subscale.^1^ The exercise of reproductive health rights has been recognized as one of the prerequisites for sustainable development in many developing countries. Therefore, women need to be able to make decisions about their own health and reproductive rights, especially during the reproductive period.^13^

Late menarche and undergoing mammography examination among the Quilombola women in the study were factors associated with lower reproductive autonomy in the "Freedom from coercion" and in the "Total reproductive autonomy" subscales. However, it should be emphasized that due to regional and social inequities in Brazil, mammography coverage is still lacking, thus highlighting the inequalities in access to mammographic screening services. Therefore, it is necessary to understand the economic and social vulnerabilities that affect access to breast cancer screening and to recognize the need for strengthening women's health policies, as well as health education initiatives.^14^

The use of healthcare services is another factor that can influence the participation of women in breast cancer control actions. Therefore, access to mammography, the main early detection examination, is not equal among Brazilian women.^15^In this context, several factors hinder the health of Quilombola communities, whether due to geographical isolation, low levels of education, limited access to healthcare services, patriarchy, or racial and gender inequalities.

The health situation is not limited to the health-disease dichotomy but encompasses various aspects of life, especially the social condition of individuals. Female vulnerability and difficulty in accessing more remote areas such as rural zones are factors that contribute to the ineffective coverage of the female population by policies for comprehensive women's health care.^16^ Thus, given the pronounced interference between racial relations and vulnerability to care, education, health information, and, primarily, the distance of vulnerable communities from the reach of comprehensiveness and equity, there is a need to promote practical health promotion strategies linked to these Quilombola populations and to integrate, especially, the Nursing team, as it is the category that is most in contact with the population within healthcare services and is responsible for the direct care of women at all stages of their life cycle.^17^

The ways in which racial relations are shaped make the Black population more vulnerable and tend to hinder their access to healthcare services. Accordingly, it is necessary to observe the health of Black women from an ethnic-racial perspective and to understand that racism can be considered a social determinant and can directly intervene in the health-disease process.^18^ Therefore, it is necessary to understand the reality of the health of these communities to provide support for adequate and effective planning of sexual and reproductive health actions, allowing these women to achieve their reproductive intentions.

## Conclusion

The results of this study reinforce that freedom in deciding reproductive choices promotes safe and satisfactory sexual life. In this sense, reproductive autonomy is positively associated with decision-making about healthcare. It should be highlighted that improvement in access to sexual and reproductive rights can ensure women's reproductive well-being and strengthen decision-making about their own health. Geographical isolation, the COVID-19 pandemic, and the scarcity of previous studies were the main limitations of this study but were not impediments to conducting the research.

Differences in sexual and reproductive patterns reflect cultural influences, socioeconomic factors, and access to healthcare services. The association of social determinants of health such as marital status, menarche, education, and age interferes with women's reproductive choices, implying risks to sexual and reproductive health. The Quilombola women in this study showed high reproductive autonomy, especially in the Decision-Making and Freedom from coercion subscales. It was found that marital status interfered with reproductive autonomy, as single or unpartnered Quilombola women showed higher overall reproductive autonomy compared to married or partnered women. Late menarche and undergoing mammography examination were factors associated with lower reproductive autonomy in the "Freedom from coercion" and "Total reproductive autonomy" subscales. 

Black and mixed-race women are subject to greater vulnerability, so it is important and necessary to identify racial biases in order to seek equity in caring for these women, allowing for differentiated assistance to this population. To improve health conditions, profound changes in economic patterns are necessary, as well as intensifying social and healthcare public policies. In the field of Nursing, research like this enables the expansion of knowledge about sexual and reproductive rights and how they are experienced in a specific population, breaking the fragmentation of care offered and providing qualified, humanized practice that respects the specificities of Quilombola women, thereby contributing to effective reproductive planning and reducing the number of unwanted pregnancies, complications resulting from the pregnancy and postpartum period, illegal abortions, and maternal and infant mortality.
